# Supplementary material for: From sequence to enzyme mechanism using multi-label machine learning
Source: BMC Bioinformatics. 2014 May 19;15:150. doi: 10.1186/1471-2105-15-150 (PMC4229970; doi:10.1186/1471-2105-15-150)
Supplement: Additional file 2 — Java code of ml2db. Additional file ml2db_code.tar.gz contains the Java source code to run the multi-label machine learning experiments and save the results to database. The code’s Javadoc is included. [file 1471-2105-15-150-S2.zip › additional file 2/ml2db/utils/doc/uk/ac/ed/inf/utils/database/DbCreator.html]

DbCreator


---


|  |  |  |
| --- | --- | --- |
| **Overview** \| **Package** \| **Class** \| **Use** \| **Tree** \| **Deprecated** \| **Index** \| **Help** | |  |
| **PREV CLASS**   **NEXT CLASS** | **FRAMES**    **NO FRAMES**     **All Classes** |  |
| SUMMARY: NESTED \| FIELD \| CONSTR \| METHOD | DETAIL: FIELD \| CONSTR \| METHOD |  |


---


## uk.ac.ed.inf.utils.database Class DbCreator

```
java.lang.Object
  uk.ac.ed.inf.utils.database.DbManaged
      uk.ac.ed.inf.utils.database.DbCreator
```

---

``` public class DbCreator extends DbManaged ```

Class

**Version:**
:   20 Feb 2009

**Author:**
:   Luna De Ferrari luna.deferrari-at-ed.ac.uk

---

**Constructor Summary**

`DbCreator(DbManager dbManager)`


| **Method Summary** | |
| --- | --- |
| `void` | `createTables()` |
| `boolean` | `dropTable(java.lang.String tableName)`             Drops the database table. |
| `boolean` | `generateTable(java.lang.String tableName, java.util.Vector<TableColumn> columns)` |
| `java.lang.String` | `getSqlToDropTable(java.lang.String tableName)`             Get sql command to drop table |

**Methods inherited from class uk.ac.ed.inf.utils.database.DbManaged**

`getDbConnection, getDbManager`

**Methods inherited from class java.lang.Object**

`equals, getClass, hashCode, notify, notifyAll, toString, wait, wait, wait`

**Constructor Detail**

### DbCreator

```
public DbCreator(DbManager dbManager)
```


**Method Detail**

### createTables

```
public void createTables()
```

---


### dropTable

```
public boolean dropTable(java.lang.String tableName)
```

:   Drops the database table. Beware! risk of data loss. Better to rename the
    old table and relaunch than to drop the table.

    :   **Returns:**: true if the table does not exist in the database anymore

---


### generateTable

```
public boolean generateTable(java.lang.String tableName,
                             java.util.Vector<TableColumn> columns)
```

---


### getSqlToDropTable

```
public java.lang.String getSqlToDropTable(java.lang.String tableName)
```

:   Get sql command to drop table

    :   **Returns:**: the drop table SQL command


---


|  |  |  |
| --- | --- | --- |
| **Overview** \| **Package** \| **Class** \| **Use** \| **Tree** \| **Deprecated** \| **Index** \| **Help** | |  |
| **PREV CLASS**   **NEXT CLASS** | **FRAMES**    **NO FRAMES**     **All Classes** |  |
| SUMMARY: NESTED \| FIELD \| CONSTR \| METHOD | DETAIL: FIELD \| CONSTR \| METHOD |  |


---
